# Separation and Characterization of Plastic Waste Packaging Contaminated with Food Residues

**DOI:** 10.3390/polym15132943

**Published:** 2023-07-04

**Authors:** Svetlana Tretsiakova-McNally, Helen Lubarsky, Ashlene Vennard, Paul Cairns, Charlie Farrell, Paul Joseph, Malavika Arun, Ian Harvey, John Harrison, Ali Nadjai

**Affiliations:** 1 Belfast School of Architecture and the Built Environment, Ulster University, Belfast Campus, York Street, Belfast BT15 1AP, UK; 2 South West College, Cookstown Campus, Cookstown BT80 8DN, UK; 3 Institute of Sustainable Industries and Liveable Cities, Victoria University, P.O. Box 14428, Melbourne, VIC 8001, Australia; paul.joseph@vu.edu.au (P.J.);; 4 Granville EcoPark, Dungannon BT70 1NJ, UK

**Keywords:** plastic recycling, plastic packaging, microplastics, polymer degradation, pyrolysis

## Abstract

In this paper, we present the development of a novel processing technology to tackle hard-to-recycle plastic packaging waste contaminated with food residues. The proof-of-concept (POC) technology can effectively separate food residual amounts from plastic waste materials to a level acceptable for further re-use or recycling of the plastic packaging. To assess this technology, we have conducted spectroscopic, thermal, and calorimetric characterizations of the obtained fractions, such as cleaned mixed plastics (CMP), food waste with mixed plastics (FWMP), and a mixture of microplastics (MP). The analyses were carried out with the aid of Fourier-Transform Infrared spectroscopy (FT-IR), Thermo-Gravimetric Analysis (TGA), Microcone Combustion Calorimetry (MCC), and ‘bomb’ calorimetry. The highest ratio of CMP to FWMP and the lowest amount of MP were obtained utilizing 700 rpm blade rotational speed and 15 s residence time of contaminated plastics in a cutting mill chamber. The plastics were freed from food contamination by 93–97%, which highlights a strong potential of the POC as a solution for ‘dry-cleaning’ of similar wastes on a larger scale. The main components of the CMP fraction were low-density polyethylene (LDPE), polypropylene (PP), and polyethylene terephthalate (PET), which are recyclable plastics. The knowledge and understanding of thermal degradation behaviours and calorimetric attributes of separated fractions, determined in this study, are essential in informing the industrial players using pyrolysis as a technique for recycling plastics.

## 1. Introduction

High amounts of food wastage are a global concern [1]. Along with the environmental, social, and economic impacts of food waste, one major challenge associated with food waste is the plastic packaging that the food is contained within. One of the routes that food contact waste can take in the end-of-life management is landfilling, both directly and indirectly, via processes such as anaerobic digestion (AD). Reducing the amounts of plastic packaging waste going into landfill via AD and enhancing the re-use and recycling of plastic materials are the most critical issues for the waste processing industry today.

In the UK alone, there are approximately 115 AD plants that receive food waste as their feedstock for renewable energy production [2]. At an AD plant, the incoming packaged food waste normally undergoes a mechanical separation in a de-packaging unit. Although this equipment separates a substantial amount of the food waste from the plastic fragments, it also heavily contaminates the packaging with organic matter. An example of the scale of this problem is an AD facility in Northern Ireland that processes around 100,000 tonnes of food waste annually, with around 8000 tonnes of plastic packaging waste going directly into landfill, or for processing into refuse derived fuel (RDF). There is a potential to optimise this process by cleaning the packaging as contaminated plastics usually are not suitable for reprocessing [3]. For both mechanical and chemical recycling, it is essential that a cleaning step has taken place. The process may include washing, with either hot or cold water, with added detergents based on caustic agents [4]. Washing with water can be costly due to the need for specialist washing facilities and, also, the need for further water treatment after plastics have been washed. Moreover, it can lack efficiency as certain contaminants cannot always be removed [4,5]. Additionally, the process can be complex, requiring four or five stages, and with the addition of media as well as longer ‘cleaning’ times [5].

Preliminary results, as part of the RE:Solve project funded by ECOSURETY, indicated that significant volumes of water (40 L/kg packaging) would be required to clean the packaging to an acceptable level for further reuse or recycling. These figures were similar to the findings of Hopewell et al., who reported that the amount of consumed water required for washing different types of rigid plastics was 32,000 L/tonne for high density polyethylene (HDPE), 43,000 L/tonne for polypropylene (PP), 66,000 L/tonne for polyethylene terephthalate (PET), and 140,000 L/tonne for polystyrene (PS), respectively [6].

The methods of ‘dry-cleaning’ of plastic waste using relatively expensive agents such as supercritical carbon dioxide, laser, and dry ice are summarised by Xia and Zhang [7]. However, the literature sources on affordable, simple, and water-free cleaning technologies are somewhat limited [5,7,8]. Song et al. employed a two-step process involving cylindrical agitation combined with an air jet cleaning of plastic waste to achieve a cleaning performance comparable with water washing [8]. To the best of authors’ knowledge, there are no recent reports on inexpensive ‘dry cleaning’ and separation of hard-to-recycle plastic packaging waste, originated from the AD facilities and heavily contaminated with food residues. Moreover, an important issue of microplastics generated during recycling processes, which is often overlooked by researchers and industry, was considered in this study.

The aim of the current study was to develop a proof-of-concept (POC) to tackle the issue of contaminated plastic packaging originated from the food waste management sector, eliminating the need for large volumes of water in a typical cleaning process. This was achieved through spectroscopic and thermal assessments of the contaminated plastic packaging, followed by development of a novel, single-step cleaning process capable of producing plastic feedstock, from food contaminated AD waste, with a degree of cleanliness above 95%, and without the use of water. Another goal of the project was to suggest routes for recycling of the cleaned and separated plastic feedstock, which has previously undergone processing in the POC, targeting R6–R9 strategies in the 9R circular economy framework [9].

The developed process for cleaning and separating plastic packaging waste resulted in two streams: Cleaned Mixed Plastics (CMP) with high level of fragments cleanliness and Food Waste with Mixed Plastics (FWMP) with particle sizes less than 2 mm. The sizes of plastic pieces within the CMP fraction varied from several cm to less than 1 mm as it contained a certain amount of microplastics (MP). The MP were isolated from CMP, for further investigation, by sieving into: MP with sizes ranging from 5.6 to 1.0 mm (labelled as larger MP) and less than 1.0 mm (labelled as smaller MP).

In this paper, spectroscopic, thermal, and calorimetric characterization of the obtained plastic fractions, with and without adhered food waste, is presented. The efficacy of the proposed process as well as investigating the production of associated microplastics are also discussed here. An additional engineering design work carried out on the POC with a view of improving the process by enhancing the extraction of plastics from the cutting mill chamber will be discussed in a follow-on publication.

## 2. Materials and Methods

### 2.1. Plastic Waste Contaminated with Organic/Food Residues

**Figure 1:**
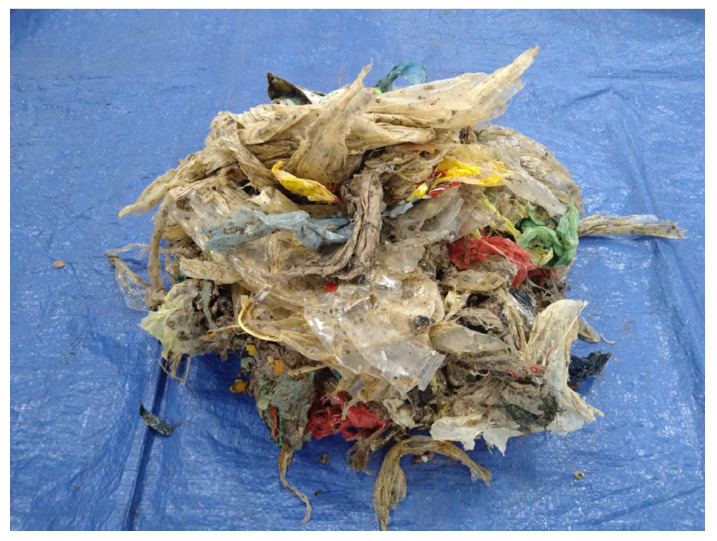
An example of plastic packaging waste as received.

For the trials used in the current project, plastic packaging waste contaminated with food residues was used. This was collected from a local Anaerobic Digestion (AD) facility that utilizes food waste as its main feedstock, which originated from regional supermarkets, restaurants, rejections from production lines, domestic food bin collections, hotels, prisons, schools, universities, and colleges. The AD plant mechanically processes this waste at the front end to remove the food from the plastic packaging in order to utilize the food only waste within the AD process. Mechanical equipment is employed to separate a substantial amount of the food waste from the packaging, which is mainly plastic. However, this process can also leave the plastic packaging heavily contaminated with food residues adhering to it, making it hard to recycle. Two batches of contaminated plastic packaging waste were received from an AD facility (Dungannon, Northern Ireland, UK) on two separate days (13 and 10 kg, respectively). From these batches of collected waste, contaminated plastic packaging was manually retrieved, weighed out (approximately 200–300 g of wet materials), and placed on trays. Then, these samples were dried in a fan-assisted oven as described below in Section 2.3. Figure 1 shows an example of the waste.

### 2.2. Processing Samples through a Cutting Mill, POC

**Scheme 1:**
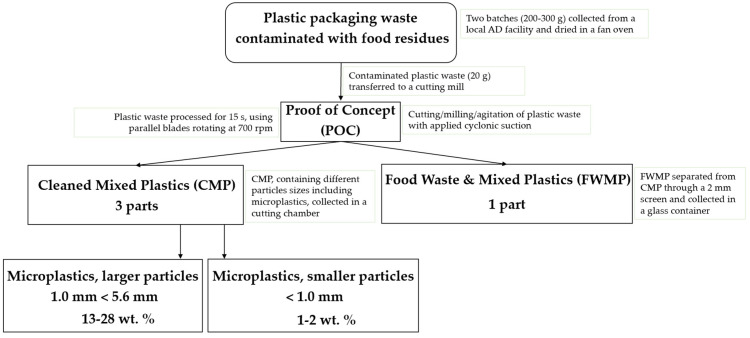
The CMP and FWMP fractions resulted from the application of the POC machine, trial 2a.

Processing of dried waste plastic samples was performed using a Retsch SM 300 cutting mill (Retsch GmbH Retsch-Allee 1-5 42781 Haan, Germany), combined with a cyclonic separator (Retsch GmbH Retsch-Allee 1-5 42781 Haan, Germany), and a vacuum extraction unit (Nilfisk GD 930S2, 1000W, Szigetszentmiklós, ÁTI Sziget Ipari Park hrsz 12001/76-77 N 47.33426 E 19.01570, 2310, Hungary), which provided the suction required during cyclonic separation. The cutting mill consisted of a hopper, a main cutting chamber, a cyclonic separator, and a glass collection container. The cutting mechanism within the chamber comprised of a rotor (with either three or six cutting blades) and three fixed cutting bars, attached on the internal chamber wall, which were able to reduce particles sizes by shearing and cutting forces. The cutting mill had a variable speed drive, allowing the speed of the rotor blade to be controlled between 700 to 3000 rotations per min (700–3000 rpm). The relatively large pieces (≤10 × 10 cm) of dried plastics, which entered the main chamber (via the hopper), experienced a cleaning effect due to the mechanical agitation and shear and cutting forces exerted via the rotor blades and fixed blades within the chamber. The simultaneous cyclonic separation extracted organic (i.e., food) particles along with the smallest cut plastic pieces, i.e., microplastics, through a 2 mm screen into a glass container, leaving behind the volume of cleaned plastic materials in the cutting chamber. The sample, collected in the glass container, was labelled as Food Waste and Mixed Plastics (FWMP). The fraction left behind in the chamber was named as Cleaned Mixed Plastics (CMP), which has also been reduced in size due to the cutting effect within the chamber (Scheme 1).

**Figure 2:**
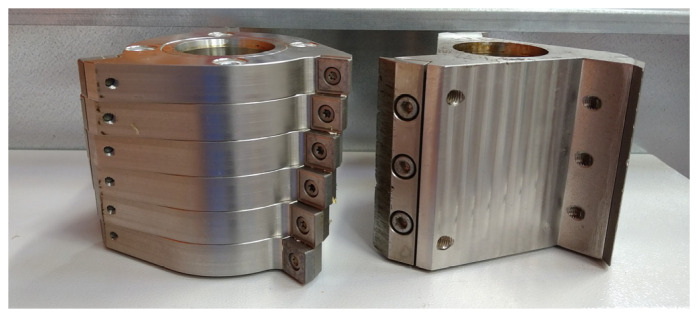
Types of blades used in the POC: offset blade (**left**), parallel blade (**right**).

Following drying, samples from the AD facility were mixed randomly, weighed, and fed into the hopper of the cutting mill. The cutting mill was switched on and a rotor speed (rpm) was set, and the cyclonic suction unit was switched on. Next, the dried plastic samples were fed into the cutting chamber and processed according to the conditions described in Table 1, which shows the rotor speed (rpm), the rotor blade type, and sample loading (g) and residence time of plastic materials in the chamber (s). Two blade types were used throughout the trials: parallel and offset (Figure 2). Preliminary tests to determine the optimum residence time of plastic fragments in the mill chamber were carried out, and 15 s and 30 s were chosen as the upper and lower limits. Following the processing, for each trial, the samples of CMP were manually collected from the cutting chamber, whilst the corresponding samples of FWMP were retrieved from the glass vessel. All the samples were weighed to determine the proportion of CMP and FWMP (in wt. %) obtained in each processing trial. The standard error in these evaluations varied from 0.5 to 2.5%. The obtained CMP and FWMP materials were placed in sealed plastic bags and stored at room temperature.

To assess the efficacy of the various mill processes, a degree of cleanliness testing was carried out on the samples in triplicates. Approximately 1 g of dried CMP material was washed with distilled water (1 part plastics/40 parts water). The stirring of the obtained mixtures was carried out using a magnetic stirrer set at 200 rpm, at room temperature. After 30 min of stirring, the mixture was allowed to settle for 5 min and then filtered, using gravity filtration, followed by drying in a fan-assisted oven, at 105 °C, until a constant mass was reached. The percentage of plastic particles freed from food residues corresponded to the cleanliness of each CMP sample.

### 2.3. Determination of Moisture Content

The moisture contents were measured by drying the samples obtained from the AD plant, in an oven as per the standard procedure [10]. The samples were weighed out first before placing them in a fan-assisted oven (Model OV/200/SS/F/DIG, Glenlab Limited, Widnes, Cheshire, UK) at 105 ± 2 °C, for 24 h. Samples were then weighed hourly until no further loss in mass was recorded, indicating a completely dried sample.

### 2.4. Separation and Sorting of CMP

Each CMP sample contained a certain amount of microplastics (MP). To determine this amount, a pre-weighed mixture taken from each sampling bag was sieved through a stacked arrangement of stainless-steel sieves with a nominal mesh aperture of 5.6 mm (No. 3.5) and 1.0 mm (No. 18). The particles with sizes less than 5.6 mm across, considered to be microplastics [11,12], were isolated. In fact, through this sieving process, two fractions were collected and weighed: MP with particles sizes higher than 1.0 mm but lower than 5.6 mm (named here as a ‘larger-size’ MP fraction), and MP with particles sizes less than 1.0 mm (named here as a ‘smaller-size’ MP fraction). The percentage of each microplastics fraction as well as the total proportion of MP within the CMP material have been calculated. The microscopic examination of MP was carried out under a dissecting microscope (Cdti STEDDY-T stereo microscope, Medline Scientific Ltd., Oxon, UK) at 40× magnification. The standard error of the MP levels ranged from 0.1 to 2.1%.

**Figure 3:**
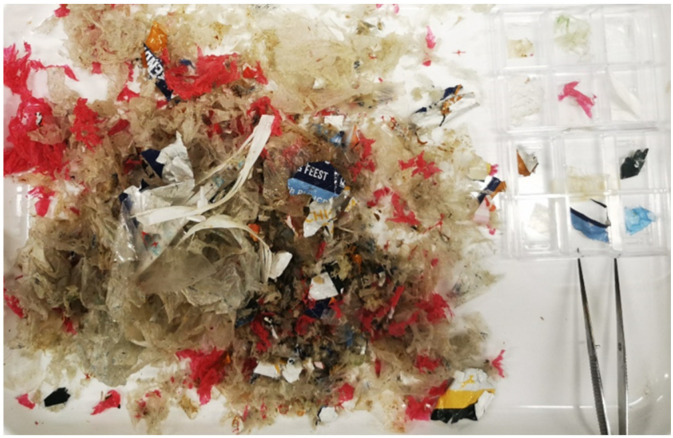
An example of separated CMP fraction according to the polymeric components.

The main portion of the CMP sample, containing particles with sizes higher than 5.6 mm, was sorted according to the types of polymers present in the mixture. From each sampling bag, using forceps, 12–14 different plastic pieces were picked according to their appearance such as colour, structure, density, and rigidity (Figure 3). The selected shredded fragments were wiped clean with a paper tissue and a Fourier-Transform Infrared (FT-IR) spectroscopic measurement was performed in the Attenuated Total Reflectance (ATR) mode to identify, or to confirm, the type of a polymeric component present in the mixture, as described in Section 2.5. Then, each mixture was taken out of a bag and manually sorted by the type of polymer. The groups containing identical polymeric pieces were weighed, and the percentage of the individual polymer fraction was calculated.

### 2.5. Fourier-Transform Infrared Spectroscopy (FT-IR)

The selected polymeric samples were characterized using Fourier-Transform Infrared (FT-IR) spectroscopy. The Fourier-Transform Infrared spectroscopic analysis in the Attenuated Total Reflectance mode (FT-IR-ATR) was carried out using a Thermo Nicolet, Nexus spectrometer (Nicolet, Green Bay, WI, USA). The spectra were collected within a wavelength range of 4000–400 cm^−1^, at a resolution of 2 cm^−1^, and have been used to confirm the type of a polymer present in the CMP fraction. The Omnic 6.1a software provided the values of the main absorption peaks, which were compared with those available in the spectra library. The identity of the polymers was confirmed when the recorded spectra had no less than 80% match to the FT-IR library data. The FT-IR spectra were run (64 scans) by placing a cleaned plastic piece (e.g., both sides cleaned from food residue) on an ATR crystal (‘diamond’) and by pressing it with a built-in pressure clamp. The ATR crystal was cleaned with ethanol after every measurement to prevent cross-contamination.

### 2.6. Thermo-Gravimetric Analysis (TGA)

The degradation behaviours of polymers and mixtures retrieved from the CMP, MP, and FWMP fractions were assessed using Thermo-Gravimetric analysis (TGA), on a Mettler Toledo instrument TGA 2 (Leicester, UK) according to BS EN ISO 11358-1: 2014 [13]. Prior to the measurements, all samples were thoroughly ground using a pestle and a mortar. Several granules of a plastic material (ca. 8–10 mg), identified through the FT-IR spectra, were placed into a ceramic crucible, and heated from 30 to 800 °C, at a heating rate of 10 °C/min in a nitrogen atmosphere, with nitrogen gas flowing at 30 mL/min rate. Thermo-Gravimetric Analysis was also carried out on the samples of mixtures from CMP, FWMP, and MP, at 50 °C/min, to compare their thermal behaviours with the MCC measurements (see Section 2.8). Given a high level of heterogeneity of the CMP and FWMP samples, the reproducibility of TGA runs was evaluated by running the test four times, and the average values of the residue left at 800 °C were calculated.

### 2.7. Wet Peroxide Oxidation (WPO)

To determine the content of residual organics (or food particles) within the microplastics (MP) portion of the CMP fraction, the ‘smaller’ particles (sizes lower than 1.0 mm) and the ‘larger’ particles (with sizes in the range from 1.0 mm to 5.6 mm) were subjected to a Wet Peroxide Oxidation (WPO) procedure described in the technical memorandum [14]. Firstly, the ‘smaller’ and ‘larger’ microplastic fractions were weighed out before applying the WPO. These MP samples were subjected to the oxidation, in the presence of iron (II) sulphate acting as a catalyst, with a view to digesting any ingrained organic matter. The microplastic particles were found to remain unaltered during the WPO. Then, the obtained mixture of dissolved organics and MP was subjected to a density separation in a sodium chloride aqueous solution in order to isolate cleaned microplastics through floatation in a density separator. The floating microplastic solids were collected by filtration on a 0.3 mm filter paper and dried in a vacuum oven at the temperature of 40 °C until no further mass loss was recorded. The dried material was removed from the oven and weighed to determine the microplastic content free from organic residue. The retention of organics (in wt. %) ingrained into MP was determined by subtracting the mass of microplastics (after the WPO) from the mass of microplastics that contained the organics (or food) attached (before the WPO) divided by the initial mass of microplastics prior to the application of WPO. The standard error varied from 2.1 to 4.5%.

### 2.8. Calorimetric Studies

‘Bomb’ calorimetric runs were performed using a Parr 6200 isoperibol calorimeter (Parr Instrument Company, Moline, IL, USA) to determine the calorific values of the CMP and FWMP in accordance with BS EN ISO 18125:2017 [15]. The measurements were conducted on the samples, in the form of a pellet, weighing ca. 0.5 g for CMP and ca. 1 g for FWMP. The ‘bomb’ was filled with oxygen up to a pressure of 31 bars and ignited. For each sample, duplicate runs were performed, and the average values are presented.

Microcone Combustion Calorimetry (MCC) measurements were performed using a Fire Testing Technology Ltd. (Gosport, UK) microcone combustion calorimeter according to ASTM D7309. For each run, an accurately weighed sample was firstly heated to about 900 °C at a heating rate of ca. 60 °C/min, in a stream of nitrogen. The thermal degradation products were collected and then mixed with a stream of air prior to entering a combustion chamber maintained at 900 °C. All the tests were run in triplicates, and the average values are reported.

## 3. Results and Discussion

### 3.1. Cleaned Mixed Plastics (CMP) and Food Waste with Mixed Plastics (FWMP)

**Figure 4:**
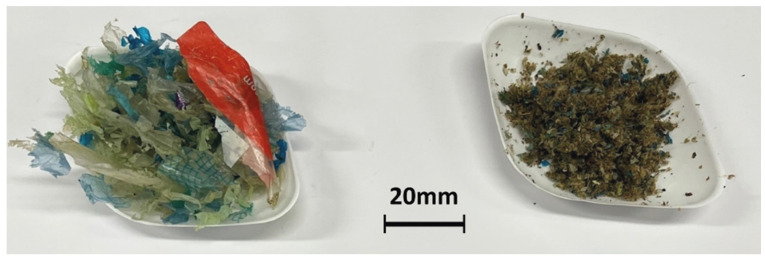
Samples obtained after the milling trial 2b: CMP (**left**) and FWMP (**right**).

After the cutting, milling, and physical agitation of dried plastics in the POC machine, as described in Section 2.2, two waste streams were obtained after each milling trial, ‘Cleaned Mixed Plastics’ (CMP) and ‘Food Waste and Mixed Plastics’ (FWMP), as shown in Figure 4. The relative amounts (in wt. %) of CMP, FWMP, and MP for each trial are reported in Table 2. The highest ratio of CMP to FWMP was achieved in the trial 2a, 75.2 wt. % of CMP/23.8 wt. % of FWMP. The CMP fraction contained a certain amount of microplastics (MP), which were isolated by sieving. The lowest amount of MP was found in the CMP samples from the trial 2a. It was not possible to separate out and collect microplastics within the FWMP fraction as the particles were found to be very ‘sticky’ and relatively small; however, the content of MP was determined through the Wet Peroxide Oxidation (WPO) procedure. This will be discussed later in the text. The moisture content of the CMP samples varied from 2.6 to 3.1 wt. % and of the FWMP samples—from 4.7 to 6.0 wt. %, respectively. The calorific values, determined through the ‘bomb’ calorimetry, ranged from 32.2 to 35.3 kJ/g for the CMP samples and from 24.7 to 24.9 kJ/g for the FWMP samples.

The impact of four different parameters of the POC was tested including the type of blade, the blade rotation speed, the loading, and the time of processing of contaminated plastics (Table 2). A sample loading size of 40 g was chosen as an arbitrary starting value for trials 1a and 1b. The rotation of blades at 1300 rpm was chosen as the starting and upper processing speed as previous tests using more than 1300 rpm resulted in a high percentage of very small plastic fragments within both CMP and FWMP fractions. Following processing in the mill for 15 s and 30 s (samples 1a and 1b, respectively), at 1300 rpm, the CMP was collected and visually inspected. The CMP was cut into small fragments, and upon the visual observation, contamination appeared to be mostly removed. Subsequently, cleanliness testing confirmed that samples 1a and 1b were 94.8% and 93.2% free from food contamination (Table 2). This confirmed that the proposed process was relatively effective at removing the dried organic matter from the dried plastic pieces.

During trials 1a and 1b, however, the sample loading of 40 g was found to be too large for the chamber, leading to clogging and inefficient rotation of the blades; a noise was emitted that was not considered a normal mill function. Therefore, it was decided to half this amount for the subsequent trials to remove any potential of damaging the rotor mechanism during processing caused by overfilling the chamber. The samples obtained through 1c and 1d trials were a repeat of 1a and 1b, using the same rotational speed and residence time conditions but with a smaller sample loading of 20 g to ensure optimum operation of blades within the chamber. Following the selected processing times, the CMP samples 1c and 1d visually appeared to be mostly free of contamination. Cleanliness testing confirmed this as 1c and 1d were 93.5 and 95.9% free of contamination, respectively (Table 2). The samples were cut to small-sized fragments of approximately 3–12 mm across during processing.

The samples 2a and 2b were processed on the parallel blades rotating at the lowest speed (700 rpm) the mill was capable of, as a direct comparison to 1c and 1d trials, to give an indication if the application of a lower speed would clean the material as effectively as a higher one. Following processing, the CMP samples 2a and 2b were observed to contain slightly larger plastic pieces than those samples processed at higher rpm. The cleanliness of the samples 2a and 2b demonstrated that they were 93.4% and 97.3% free of contamination. This result was promising as it showed that using a slower rotation—which potentially had a lower energy consumption of the mill—resulted in a comparable efficiency of food contamination removal. A full energy assessment was outside of the scope of this work. It also showed that by reducing the rpm, the size of cut fragments of the output material can vary, due to the CMP processed at 700 rpm being a slightly larger size, when compared to CMP processed at 1300 rpm.

An alternative six-discs blade (offset) was used to trial if this setup could clean the contaminated plastics effectively, and if it had an impact on the amount of microplastics generated. The offset blade was used for trials 3a–3d, repeating the number of rpm and residence times as described above and in Table 2. Following processing, the CMP for 3a–3d trials were found to have similar, smaller particle sizes and visually looked to be free from contamination. The cleanliness results showed that samples 3a, 3b, 3c, and 3d were 94.2%, 93.3%, 91.3%, and 89.3% free of contamination, respectively, which was slightly lower than the values obtained for the samples using the parallel blade. These results indicated that changing the blade type may reduce the cleaning effect within the chamber and, therefore, may negatively impact how much organic residue is removed.

Upon observation, for every trial sample, it was found that the FWMP contained very small particles of the mixed microplastics. The data obtained through the WPO and microscopy suggested that, on average, FWMP samples contained from 63 to 82% of organic matter and from 18 to 27% of microplastics with sizes less than 1 mm. When the higher rotation speed was applied, fewer microplastics were found in the FWMP: 15.1 ± 1.0% for 1300 rpm vs. 24.5 ± 4% for 700 rpm. Other variables such as blade type and residence time had a negligible impact on the composition of the FWMP.

The proposed POC process, utilizing a rotational speed of 700 rpm and a short residence time of 15 s (trial 2a), produced plastics that were 93.4% free of food contamination. This is a very fast processing time when compared to a recent study [5], where the optimum cleaning rate of 94% was achieved through 45 min of cleaning/agitation, which took place using a sand media. This highlights the strong potential of the POC discussed here as a solution for ‘dry-cleaning’ (i.e., without water) of contaminated plastics on a larger scale, due to the high cleaning efficiency and short processing times. Additionally, the cutting mechanism, which forms part of the overall cleaning process, is effective in helping to remove contamination, as residues are not trapped in folds or other parts of the packaging, which can occur in other dry-cleaning processes [4]. Moreover, engineering design work (not detailed in this study) was used to modify and enhance the original POC. Trials were carried out using 700 rpm at 15 and 30 s, and both samples were found to be 99% free of contamination, higher than achieved in [8].

### 3.2. Components of the CMP Fraction

**Figure 5:**
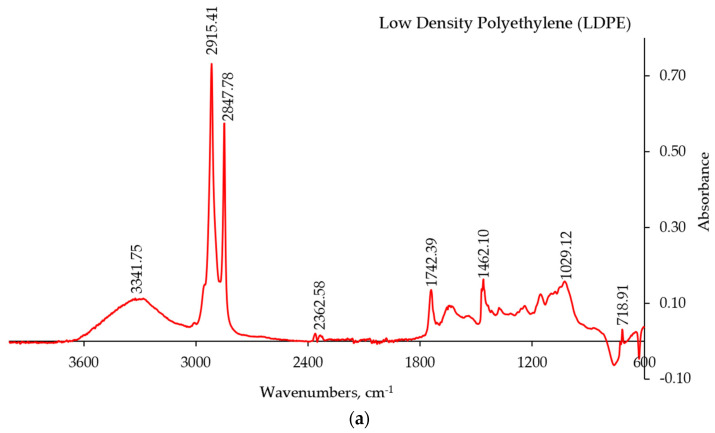

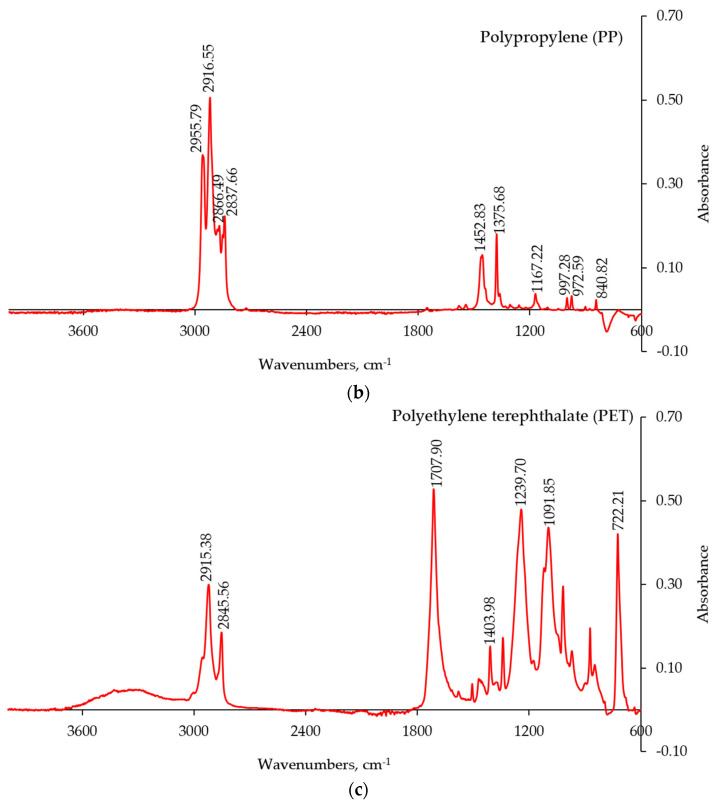
FT-IR spectra of individual polymers found in the CMP fraction: LDPE (**a**), PP (**b**), and PET (**c**).

The FT-IR-ATR spectra of the major plastic components in the CMP stream are presented in Figure 5. The obtained IR spectra were compared with the spectral data of pure polymers [16,17] and with the data from the FT-IR library available as part of the Omnic software package. The most frequently encountered individual components of the CMP were low density polyethylene (LDPE, Figure 5a), polypropylene (PP, Figure 5b), and polyethylene terephthalate (PET, Figure 5c).

The other types of polymers were also identified through the FT-IR technique, although they were present in much smaller amounts and occurred less frequently in the mixture. These components included: polyvinylchloride (PVC), polyamide (PA), polystyrene (PS), and polyurethane (PUR). Some copolymers such poly(ethylene-*co*-propylene) and more complex composites were also detected using the FT-IR spectroscopy.

**Figure 6:**
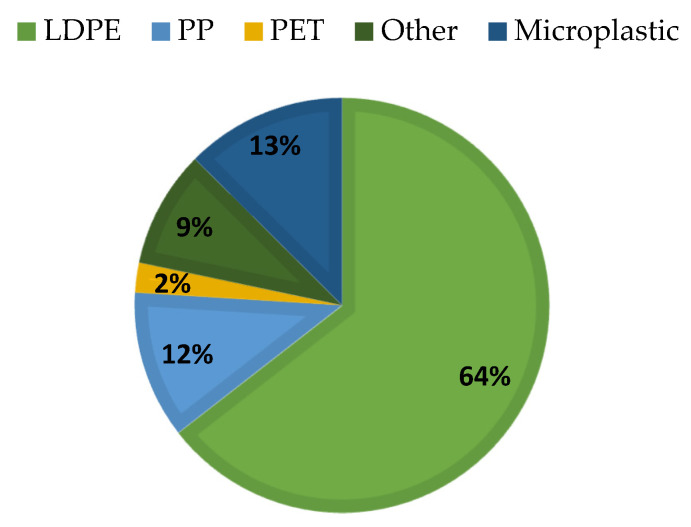
The average composition of the CMP stream.

The individual polymers retrieved from the CMP and identified with the aid of FT-IR-ATR were sorted into groups. Each group containing the identical polymer pieces has been weighed out and the percentage (wt. %) of each individual plastic type in the total mass of CMP was calculated. The average composition of the CMP fraction is shown in Figure 6. The main constituents of the CMP were LDPE (64 ± 7 wt. %), PP (12 ± 3 wt. %), PET (2 ± 0.6 wt. %), and the mixture of other polymers (9 ± 1 wt. %), some of which were identified with the aid of FT-IR spectra. Similar results are reported in [11].

The analysis and assessment of microplastics carried out as part of this study has not been carried out in detail elsewhere, which is a particularly novel aspect of this work. Microplastics are a prevalent problem related to reuse and recycling of mixed plastics [11]. The learnings detailed here provide a basis for improving the POC as well as informing recyclers how to utilize this fraction. The content of microplastics (MP), on average, was found to be 13 ± 3 wt. %. The microscopic examination of the MP particles suggested that they contained small (less than 3 mm in length) fibres as well as shards and fragments that were heavily coated with organic matter. There were some dark-brown coloured particles observed in the mixture of MP, which could be explained by the presence of coagulated organic matter (such as fats and oils) attached to the MP.

It was found that, on average, the largest polymer fraction in each CMP sample contained LDPE, which is similar to the findings published elsewhere [18,19]. The other polymers identified using FT-IR-ATR method in each sample were PVC, PS, PUR, and PA. The contents of the individual polymeric components within the CMP samples varied widely from one trial sample to another, but, nevertheless, it was estimated that in total between 73 and 94% of these components can be considered as recyclable [11,18,19].

The percentages of the ‘larger’ (size ranged from 1.0 mm to 5.6 mm) and ‘smaller’ (size less than 1.0 mm) microplastics within the CMP fraction were calculated and reported in Table 2. The data revealed that the percentage of ‘larger’ MP particles was, on average, ten times higher than the one determined for ‘smaller’ particles. This ratio remained relatively constant, irrespective of the processing trial (Table 2). The increase in the residence time of plastic waste (from 15 s to 30 s) led to an increased amount of the ‘larger’ microplastics (e.g., 13.3 wt. % for sample 2a and 27.6 wt. % for sample 2b). However, the processing time had a negligible impact on the amount of ‘smaller’ microplastics generated, which ranged from 1.1 to 2.3 wt. % for 20 g loading trials. The total amount of MP was more than doubled when the loading was doubled (e.g., samples 1d and 1b, or samples 1c and 1a) but the higher loading of 40 g was rejected due to the capacity restrictions of the cutting chamber. The cutting/milling at 700 rpm, irrespective of the type of blade, seemed to have a limited impact on the total amount of microplastics generated (e.g., sample 2a, parallel blades, 14.4% of MP and sample 3c, offset blades, 15.4% of MP). However, this may have been in part due to the shorter residence time of 15 s. The offset blade on average produced higher amounts of MP for samples, as shown from the total MP for samples 3a, 3b, and 3d.

The increase in the speed of blades rotation from 700 to 1300 rpm for the offset blades also resulted in the increase of the MP generated, which was more pronounced for the trials carried out at shorter processing times, 15 s. It is noteworthy here that the amount of microplastics generated can be controlled by varying the moisture content of contaminated plastics feedstock. For example, a repeat of trial 2b was carried out, using the same processing parameters but with an increase of moisture content of the input material from approx. 1.2 to 7.0 wt. %, which led to more than 50% reduction in the total amount of microplastics produced, i.e., from 28.7% to 12.8%, respectively. Further recommendation to reduce the volume of MP present in the CMP fraction is to place below the main chamber of the mill a screen with the mesh size higher than 2 mm but lower than 5 mm. The lowest amount of total microplastics was generated in the case of CMP sample trial 2a (14.4 wt. % in total), which was achieved by processing 20 g of waste plastics on the parallel blades running at 700 rpm for 15 s. A repeat of 2a and 2b processing parameters again showed the 2a runs produced less MP than 2b trials with 19.3% for 2a repeat and 23.9 for 2b repeat; therefore, these parameters were selected as the optimum parameters for the POC.

### 3.3. The Retention of Organic (Food) Matter by MP

The visual inspection and microscopic examination through a dissection microscope suggested that a higher level of organics (food residue) is retained by, or ingrained in, microplastics particles of the CMP stream. Both microplastics fractions with ‘larger’ and ‘smaller’ particles were subjected to the WPO process that digested any organic matter present, leaving the microplastic fragments intact. The obtained values of organics retention for each sample/trial are presented in Table 3.

The organics retention on the ‘smaller’ particles was in the range between 28.7 and 68.1% and, on average, was three times higher than the one calculated for the ‘larger’ particles of microplastics, which ranged from 6.8 to 21.2%. A comparison of samples collected through the milling trials showed that the loading did not drastically affect the levels of organics attached to the ‘larger’ microplastics (e.g., 15.9% for samples 1b and 1d). However, the level of organic matter ingrained in the ‘smaller’ microplastic particles more than doubled when the loading was halved (Table 3). The longer time of processing seemed to reduce the percentage of organics attached to the microplastics for the samples collected from the offset blades (e.g., samples 3a and 3b). The impact of the type of blade and its rotation speed was not clear from the data we have obtained. The lowest retention of organics was found for the sample collected in trial 2a, selected for the POC (see Section 3.2).

### 3.4. Pyrolysis Studies of CMP, FWMP, and MP Fractions

Thermochemical (or chemical) recycling, achieved via pyrolysis and closely associated processes, currently, is the most practical solution for food contact packaging and a viable alternative to mechanical recycling [11,20,21]. It can process heterogeneous mixed plastic waste freed from food residues, for example, the CMP obtained in this study. Through this recycling route, polymers undergo thermochemical decomposition, producing low molecular mass compounds, which can be used to produce polymers again and/or to manufacture fuels or useful chemicals [20,21,22]. To optimise such technology, deep knowledge and understanding of how the waste packaging plastics behave at elevated temperatures are essential [11]. With this in mind, we have carried out Thermo-Gravimetric Analysis (TGA) of the isolated fractions.

**Figure 7:**
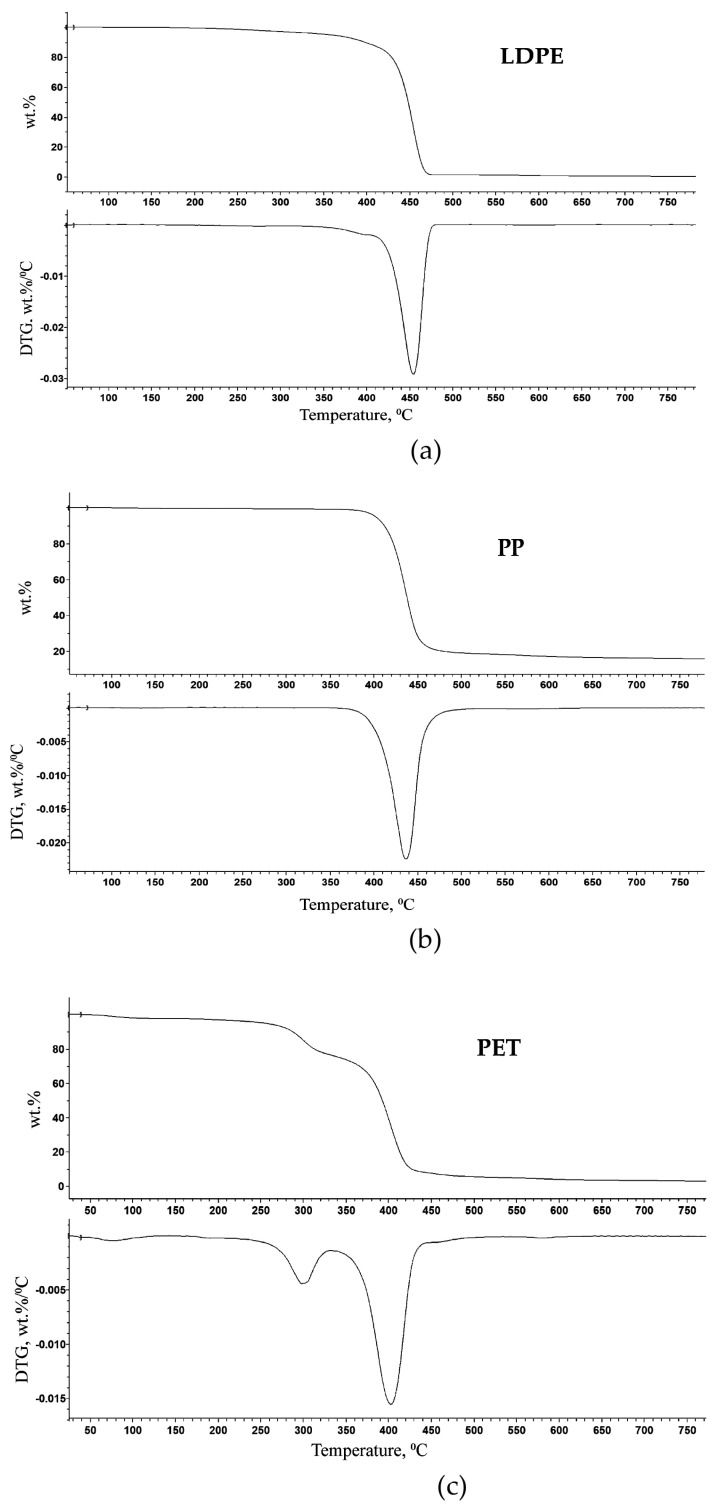
TG (**top**) and DTG (**bottom**) curves recorded for the identified polymers of LDPE (**a**), PP (**b**), and PET (**c**).

The TGA was performed to study thermal decomposition behaviours of CMP, MP, and FWMP in the inert atmosphere (i.e., pyrolysis) and to determine the temperatures corresponding to the thermal degradation of individual polymeric components or their mixtures. The TG and DTG curves were recorded, at a heating rate of 10 °C/min, on the samples of some individual polymers identified earlier through the FT-IR. The onset temperature (T_o_) corresponded to the point at which the mass loss commenced. The temperature of the highest rate of the mass loss (T_max_) was evaluated from the peak value(s) registered on the DTG curves. The T_o_ values for LDPE and PP were found to be around 400–420 °C, indicating that thermal decomposition of these individual polymeric components started in this region. The thermal degradation of samples of PET commenced at a lower temperature of 290 °C (Figure 7). The maximum rate of thermal degradation for each type of polymer was registered within the temperature interval of 420–490 °C as seen from a sharp and drastic step of mass loss (from 85 to 95 wt. %) for all the polymers (Figure 7a–c). LDPE and PP were characterized using single-step decomposition profiles, while PET showed a two-stage decomposition under the controlled conditions (Figure 7c). The initial degradation of PET started at 290 °C, followed by the main degradation step commencing at a higher temperature of 405 °C. The initial degradation of PET may be due to the presence of some volatile additives (e.g., colourants, plasticizers, or other fillers) used during polymerization [22,23]. Therefore, 450 °C could be taken as the optimum temperature for catalytic pyrolysis of the plastic waste [23].

**Figure 8:**
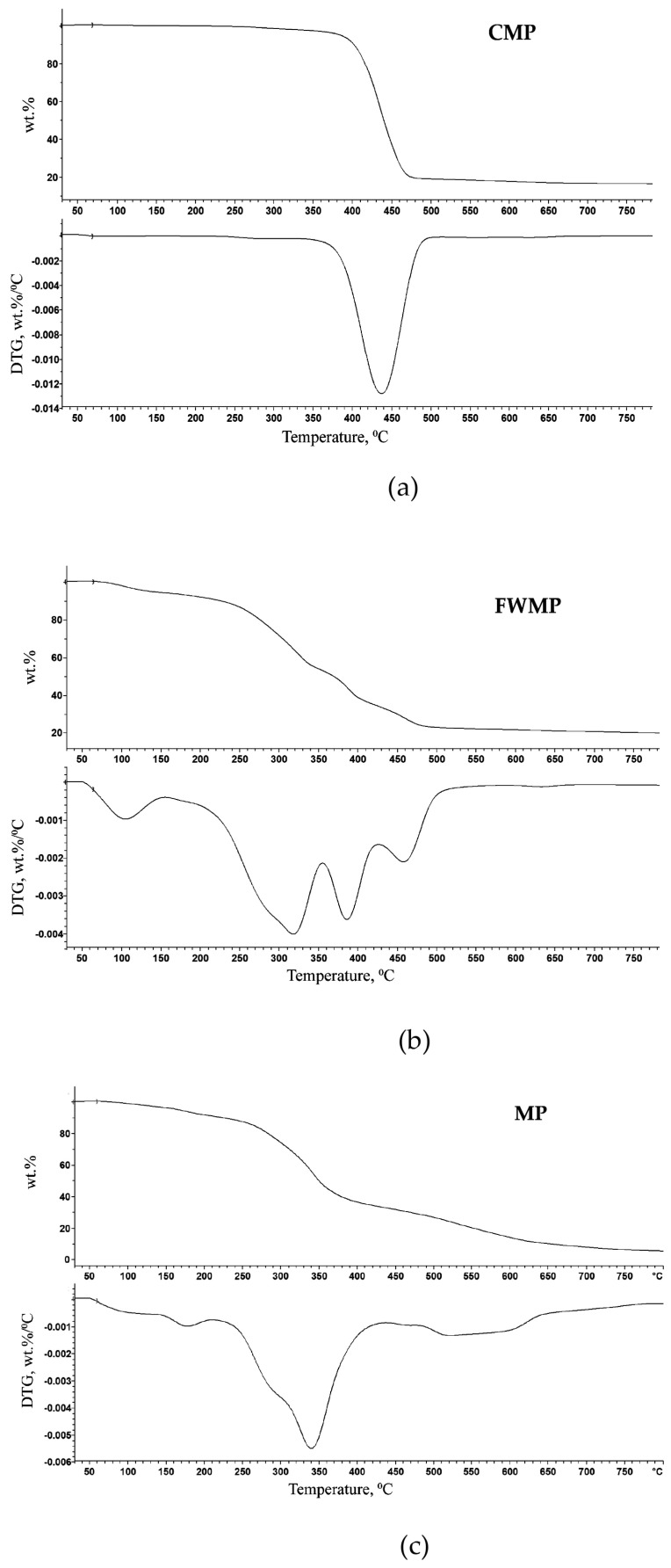
TG (**top**) and DTG (**bottom**) curves of CMP (**a**), FWMP (**b**), and MP (**c**) of sample 2a recorded at a heating rate of 50 °C/min.

As it follows from Figure 8a, showing the TG and DTG curves recorded at the heating rate of 50 °C/min, a one-step degradation commencing at about 310–320 °C was observed for the CMP sample 2a. It is highly likely that at this stage monomer(s) such as ethylene and propylene were produced. The isolation of the monomers (i.e., the precursors of polymers) would allow the regeneration of the corresponding polymers through chemical recycling. This is one of the feasible routes for recycling the CMP stream as the enhanced cleanliness level was achieved through processing of contaminated plastics on the POC. All other samples of CMP fraction demonstrated similar degradation behaviour, which was associated with the rapid loss of mass occurring within the 380–500 °C range of temperatures. The single-step decomposition can be explained by the breakage of carbon–carbon bonds undergoing the random chain scission mechanism upon heating [22]. The relatively low amount of solid residue formed by the CMP sample 2a at the end of TG measurement at 800 °C was found to be 13.6 ± 2.0 wt. %.

The TG and DTG curves recorded for the FWMP sample 2a (as seen from Figure 8b) exhibited the decomposition route occurring in five stages: first—from 50 to 100 °C, second—from 100 to 170 °C, third—from 180 to 350 °C, fourth—from 350 to 430 °C, and fifth—from 430 to 500 °C. The mass loss registered below 100 °C (stage I) can be attributed to the evaporation of surface water present in the sample. The mass loss, which could be linked to the removal of bound or intracellular water, continued gradually and relatively slowly up to the temperature point of approximately 170 °C (stage II). Then, a more rapid mass loss occurred between 180 and 350 °C (stage III) that may indicate the start of degradation of some organic compounds such as fats, lipids, carbohydrates, and oils. The possible depolymerization of synthetic components, including microplastics, is visualised by two peaks of mass loss at approximately 385 °C and 460 °C, as seen in the DTG curve (Figure 8b). These stages (IV and V) marked the polymers degradation, as described earlier in [22]. The solid residue obtained at 800 °C following thermal decomposition of the FWMP sample 2a was 19.8 ± 1.2%. It is interesting to note that the TG residue measured for the FWMP sample was slightly higher than the one measured for the CMP from the same milling trial, owing to a higher level of organic (food) residue present in the FWMP. Indeed, FWMP samples contained from 63% to 82% of organic matter as mentioned above in Section 3.1. The processes observed in the temperature interval of 350–430 °C can be associated with the decomposition of the residual food amounts, organic impurities and small particles of plastics, or additives to plastics, and a slow degradation of their large molecules. The processes commencing at approximately 430 °C can be attributed to the exothermic transformations associated with the production of pyrolysis gases from the solid residues [22].

Figure 8c shows the TG and DTG traces of MP fraction isolated from the CMP stream of 2a trial. Unexpectedly, the microplastics exhibited the multi-staged decomposition behaviour (instead of a one-step process as observed for the CMP sample shown in Figure 8a) in relatively wide temperature intervals: from 50 to 140 °C, from 150 to 210 °C, from 210 to 430 °C, and from 430 to 650 °C. It was difficult to distinguish exactly how many stages had occurred as some processes were overlapping. This type of behaviour indicates that microplastic particles, isolated by sieving of the CMP fraction, were still contaminated with the organic residual matter, which was confirmed by the data shown in Table 3. The mass loss registered in the 50–140 °C interval can be attributed to the evaporation of surface and bound water present in the sample. Similar to the TG and DTG curves of the FWMP (Figure 8b), a more rapid mass loss occurred between 210 and 430 °C that may indicate the degradation of organic impurities strongly adhered to microplastic particles as well as the generation of depolymerization products. In the region between 430 and 650 °C, several processes occurred, leading to a total mass loss of 93.5 wt. %. The final stages of thermal decomposition may correspond to the production of volatiles and charring. The amount of residue left after the completion of thermal degradation at 800 °C was 6.1 ± 1.1 wt. %.

Thermochemical conversion could be considered as a viable recycling route targeting both plastic and food waste streams when they are mixed, and this would eliminate the need for landfilling. Determining the rate of conversion of these materials and kinetic parameters associated with this will be useful in helping to scale up the conversion studies. The kinetic triplet, which refers to the activation energy, pre-exponential constant, and rate of reaction can be used in a reactor design and for a potential process modelling as these kinetic parameters are not bound by the scale.

### 3.5. Calorimetric Evaluations of Selected Samples from CMP, FWMP, and MP Fractions

Microcone combustion calorimetry (MCC), also known as Pyrolysis Combustion Flow Calorimetry, has been shown to be a very valuable small-scale technique for screening flammability of different polymeric materials [24]. In the present study, MCC was chosen as a rapid technique that requires only a few milligrams of a solid material for testing and often provides a wealth of pyrolysis-related data [25]. The technique works on the principle of oxygen consumption calorimetry, relating to the Hugget’s principle that 1 kg of consumed oxygen corresponds to 13.1 MJ of released energy for any organic material undergoing combustion. At first, the solid sample is rapidly heated to a state of controlled pyrolysis in the inert atmosphere of nitrogen, followed by a rapid oxidation at high temperatures (i.e., combustion) of the pyrolysates in the air [26,27]. The following quantities were measured: peak heat release rate (pHRR); temperature at peak heat release rate (Temp to pHRR); total heat release (THR); heat release capacity (HRC); and the percentage of the char residue. The instrument also recorded corresponding plots of the heat release rate (HRR) vs. temperature. Another useful parameter that can be calculated from THR and the char residue (ratio of the mass of the original sample to the mass of the residue: g/g) is the effective heat of combustion (EHC: kJ/g): EHC = *THR*/(1 − *Y_p_*) where *Y_p_* = (mass of the sample)/(mass of the residue)

For each run, an accurately weighed sample (ca. 25 mg) was first heated to about 900 °C at a heating rate of ca. 1 °C/s, in a stream of nitrogen. The volatile thermal degradation products, thus obtained, were then mixed with a stream of air prior to entering a combustion chamber maintained at 900 °C. All the tests were run in triplicate, and the average values are given (Table 4). The results are summarized in Table 4. The high level of heterogeneity of the samples is reflected in the relatively high standard deviation errors.

The high cleanliness level of synthetic polymers in the CMP fraction is reflected in the high amounts of total heat released and heat release capacity values found in other reports [26,27,28]. As for the samples of FWMP, these parameters were significantly lower, obviously due to the high levels of food residues. It could be also seen that the particles of MP fraction contained some level of food contamination. This is clearly visible from the HRC and EHC measured for the CMP and MP samples. For example, for the sample from 3b trial, the EHC value for the CMP was 39.2 ± 3.8 kJ/g, whilst for the MP, it was 33.6 ± 2.4 kJ/g. Generally, the measured vales of EHC correlated well with the calorific values determined for the CMP and FWMP, as reported in Section 3.1. Also, there is a reasonably good agreement between the residues found through MCC and TGA.

Due to the nature of the small plastic particles in the FWMP stream, a suggested measure is a route that allows the utilisation of both waste sources. Additionally, attempting to separate the FWMP stream further could prove difficult and would require a higher processing time and more resources/utilities in the form of electricity. Similarly, a smaller particle size means that there are less heat and mass transfer limitations when considering thermochemical conversion routes such as pyrolysis. With less heat and mass transfer limitations, this means that the thermochemical conversion process is more efficient.

With this in mind, it is the authors’ suggestion that this fraction could be trialled in a co-pyrolysis process where both waste sources are converted. Due to the organic nature of the food waste, firstly, a gas analysis would need to be completed to determine exactly which route to take. The pyrolysis process can be altered to help maximise specific products, such as lowering the heating rate and hold (residence) time to maximise solid fraction or increasing the heating rate to increase the liquid fraction. However, assuming that food waste pyrolysis would yield complex products, it is in our belief that harnessing the heat from the pyrolysis gas would be a good alternative for general heating of the water and a building at the given recycling site. This would help alleviate the need for utilities and would help offset carbon emissions that originate from electricity generated from fossil fuels. This would help harness the inherent value in these waste materials and would help contribute positively to the circular economy for these waste materials without having to utilise more energy resources and generate more emissions in the manufacture of other useful products. Finally, it also addresses the landfilling issue of both materials.

**Figure 9:**
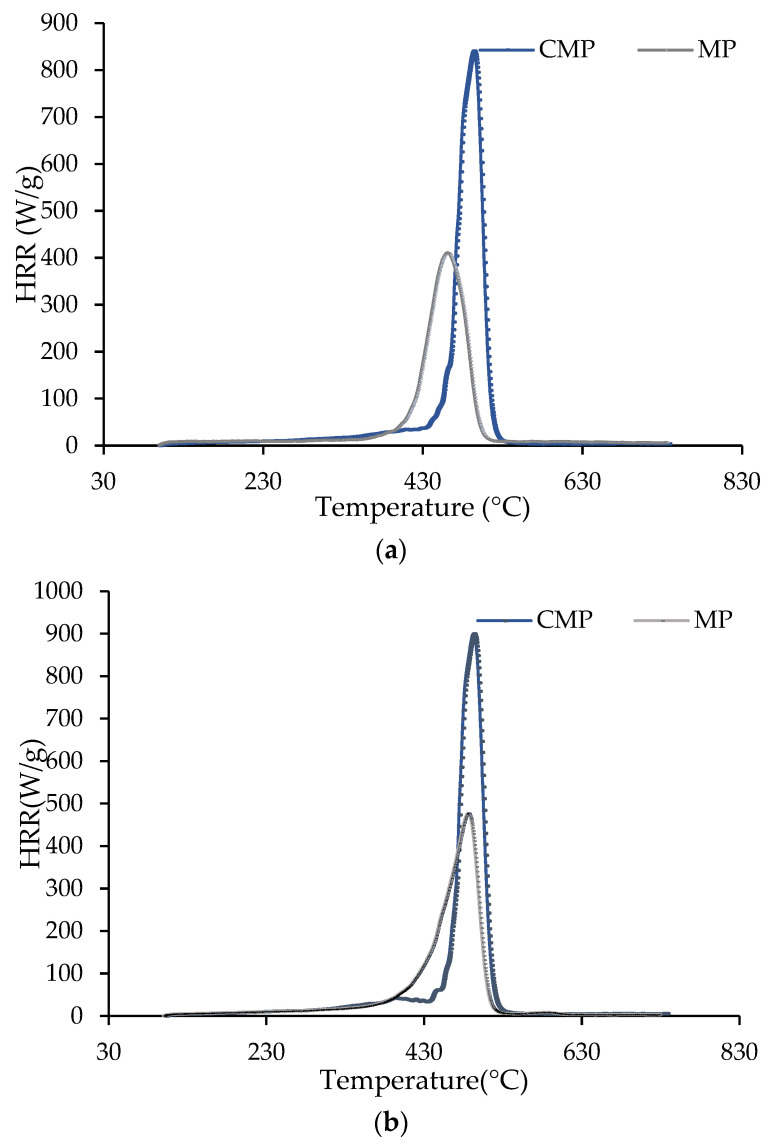
The average HRR vs. temperature for CMP and MP fractions: sample 2b (**a**) and sample 3b (**b**).

**Figure 10:**
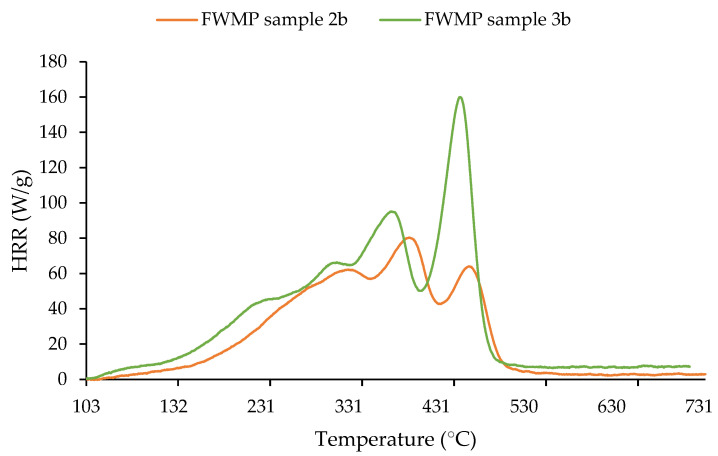
The average HRR vs. temperature for FWMP fraction.

The evolution of the HRR with temperature for the CMP, MP, and FWMP fractions of the samples collected in trials 2b and 3b are shown in Figure 9 and Figure 10. These profiles are in tune with the results from TGA, especially, regarding the temperature intervals corresponding to thermal degradation of the materials, i.e., from 380 to 520 °C for CMP and MP (Figure 9), and much wider temperature range for the FWMP samples (Figure 10). Also, the higher content of synthetic polymers (or the higher cleanliness) in the CMP can be seen from the more intense and narrower HRR peaks as opposed to the one recorded for MP ingrained with residual organics. The multi-stage decomposition, also observed from TG and DTG curves for the FWMP, is also reflected in HRR vs. temperature curves of FWMP samples (Figure 10).

## 4. Conclusions

In this study, the feasibility of a simplified, ‘dry-cleaning’ method for contaminated plastic packaging through a novel proof-of-concept (POC) process, which involved cutting, shearing, and mechanical agitation of infeed food contact plastics, has been demonstrated. It was found that food residues could be effectively removed from dried contaminated plastic packaging to achieve a degree of cleanliness above 95% via the proposed separation process, thus eliminating the need for large volumes of water. The simultaneous use of a cutting mill and a cyclonic separator produced two streams, Cleaned Mixed Plastics (CMP) and Food Waste and Mixed Plastics (FWMP). The highest ratio of CMP to FWMP, 75.2 wt. %:23.8 wt. %, was obtained in the process utilizing a relatively high blade rotational speed (700 rpm) and a short residence time (15 s). The POC discussed here provides a strong opportunity to circumvent contaminated plastic packaging waste from landfill. It was estimated that in total up to 94% of the CMP components can be considered as recyclable. The CMP produced via the prototype has several potential routes for more sustainable end-of-life management. This includes remanufacturing routes into products such as chipboard/plywood alternatives or recovery routes such as pyrolysis. Thermal degradation behaviours and calorimetric attributes of the separated fractions, reported in this paper for the first time, form an essential knowledge base that will inform the plastics recyclers utilizing pyrolysis as a main technique. The assessment of microplastics generated through the POC is a particularly novel aspect of this work. It was found that the use of the offset blade and an increase in the residence time were the main factors affecting MP generation. The lowest amount of total microplastics was produced in the case of trial 2a.The conditions selected for the POC were based on processing 20 g of waste plastics, on the parallel blades running at 700 rpm for 15 s.

## Figures and Tables

**Table 1:** Different trials and parameters of the cutting/milling process used for the POC.

Trial ID	Rotation Speed (rpm)	Time (s)	Sample Loading (g)	Blade Type
1a	1300	15	40.6	parallel
1b	1300	30	40.3	parallel
1c	1300	15	20.8	parallel
1d	1300	30	21.2	parallel
2a	700	15	20.2	parallel
2b	700	30	20.5	parallel
3a	1300	15	20.0	offset
3b	1300	30	20.7	offset
3c	700	15	20.2	offset
3d	700	30	20.6	offset

**Table 2:** Obtained sample trials and parameters of the cutting/milling process used for the POC ^1^.

Trial ID	Rotation Speed (rpm)	Time (s)	Loading (g)	Blade Type	CMP (wt. %)	FWMP (wt. %)	MP Larger Size (wt. %)	MP Smaller Size (wt. %)	Total MP (wt. %)	Cleanliness of CMP (%)
1a	1300	15	40.6	parallel	55.4	32.3	31.7	3.8	35.5	94.8
1b	1300	30	40.3	parallel	60.5	37.0	42.8	4.4	47.2	93.2
1c	1300	15	20.8	parallel	66.8	32.2	16.9	1.8	18.7	93.5
1d	1300	30	21.2	parallel	67.0	30.7	17.0	1.7	18.7	95.9
2a	700	15	20.2	parallel	75.2	23.8	13.3	1.1	14.4	93.4
2b	700	30	20.5	parallel	66.8	30.7	27.6	1.1	28.7	97.3
3a	1300	15	20.0	offset	71.5	26.0	19.3	1.5	20.8	94.2
3b	1300	30	20.7	offset	53.6	44.0	27.0	2.3	29.2	93.3
3c	700	15	20.2	offset	72.3	23.8	13.5	1.9	15.4	91.3
3d	700	30	20.6	offset	61.2	36.4	26.4	1.9	28.3	89.3

^1^ Samples 1a and 1b were not considered for POC but are shown here for completeness.

**Table 3:** The percentage of organics retained by different sizes of microplastics.

Trial ID	Rotation Speed (rpm)	Time (s)	Loading (g)	Blade Type	Retention of Organics by ‘Larger’ MP (%)	Retention of Organics by ‘Smaller’ MP (%)
1a	1300	15	40.6	parallel	13.1	42.3
1b	1300	30	40.3	parallel	15.9	31.0
1c	1300	15	20.8	parallel	6.8	66.8
1d	1300	30	21.2	parallel	15.9	68.1
2a	700	15	20.2	parallel	20.0	28.7
2b	700	30	20.5	parallel	18.8	64.4
3a	1300	15	20.0	offset	21.2	59.9
3b	1300	30	20.7	offset	14.8	44.6
3c	700	15	20.2	offset	10.5	53.3
3d	700	30	20.6	offset	10.9	53.8

**Table 4:** The results of MCC for selected samples of CMP, FWMP, and MP.

Fraction	Sample ID	HRC J/(g·K)	pHRR (W/g)	THR (kJ/g)	Temp to pHRR (°C)	EHC (kJ/g)	Residue (%)
CMP	2b	707.7 ± 113.3	705.7 ± 117.4	29.4 ± 4.9	487.2 ± 12.2	33.3 ± 7.3	10.3 ± 12.2
CMP	3b	816.5 ± 190.6	814.9 ± 186.8	34.6 ± 6.5	483.2 ± 9.6	39.2 ± 3.8	6.7 ± 8.1
MP	2b	580.7 ± 154.5	580.4 ± 154.6	28.3 ± 4.1	462.5 ± 14.2	31.6 ± 3.5	10.5 ± 5.7
MP	3b	595.5 ± 140.2	593.6 ± 139.6	30.6 ± 1.8	488.1 ± 3.9	33.6 ± 2.4	10.4 ± 1.9
FWMP	2b	131.0 ± 51.9	109.5 ± 57.8	18.0 ± 1.6	394.8 ± 78.5	21.3 ± 1.9	18.6 ± 1.7
FWMP	3b	175.3 ± 15.4	157.4 ± 9.7	21.2 ± 0.4	478.1 ± 2.9	26.2 ± 0.9	19.0 ± 1.5

## Data Availability

Data supporting this study are openly available from Ulster University’s Research Portal at https://doi.org/10.21251/ade5f23b-7a3f-4082-95cc-e38f564fe48d.
